# Long donor leukocyte telomeres raise risk of severe COVID-19 in recipients of allogeneic hematopoietic cell transplant

**DOI:** 10.3389/fimmu.2025.1524608

**Published:** 2025-04-29

**Authors:** Kyra J. W. Mendez, Tsung-Po Lai, Stephen R. Spellman, Simon Verhulst, James Anderson, Wael Saber, Shahinaz M. Gadalla, Abraham Aviv

**Affiliations:** ^1^ Division of Cancer Epidemiology and Genetics, National Cancer Institute, Bethesda, MD, United States; ^2^ Cancer Prevention Fellowship Program, National Cancer Institute, Bethesda, MD, United States; ^3^ Center of Human Development and Aging, New Jersey Medical School, Rutgers, NJ, United States; ^4^^®^ CIBMTR(Center for International Blood and Marrow Transplant Research), NMDP, Minneapolis, MN, United States; ^5^ Groningen Institute for Evolutionary Life Sciences, University of Groningen, Groningen, Netherlands; ^6^ School of Aquatic and Fishery Sciences, University of Washington, Seattle, WA, United States; ^7^ Center for International Blood and Marrow Transplant Research, Medical College of Wisconsin, Milwaukee, WI, United States

**Keywords:** allogeneic hematopoietic cell transplant, COVID-19, SARS-CoV-2, leukocyte telomere length (LTL), telomeres

## Abstract

**Introduction:**

Short leukocyte telomeres are associated with an increased risk of severe COVID-19 in the general population, likely due to a weakened T-cell response to SARS-CoV-2. This may lead to an amplified neutrophil response, causing pulmonary damage. Allogeneic hematopoietic cell transplant (HCT) offers an experimental setting to examine further the role of telomere length (TL) in COVID-19 severity, as leukocyte TL in recipients post-HCT reflects TL in donor leukocytes before HCT and SARS-CoV-2 infection.

**Methods:**

We examined the relationship between donor leukocyte TL pre-HCT and COVID-19 severity post-HCT in 87 HCT recipients hospitalized for COVID-19 between March 2020 and January 2022. Using the Telomere Shortest Length Assay (TeSLA), we measured leukocyte TL and the percentage of telomeres shorter than 3 kilobases.

**Results:**

The risk of severe COVID-19 in HCT recipients was associated with long telomeres (P=0.005) and a lower percentage of telomeres shorter than 3 kilobases (P=0.01) in donor leukocytes. Moreover, long donor leukocyte telomeres were associated with an increased risk of recipient mortality within four months after COVID-19 hospitalization (P=0.03).

**Conclusions:**

These findings suggest that long donor leukocyte telomeres may trigger an excessive neutrophil response and severe COVID-19 in allogeneic HCT recipients, potentially due to a transplant-related but TL-independent weak T-cell response.

## Introduction

In the general population, adults with short leukocyte telomeres are at a higher risk for severe Coronavirus Disease 2019 (COVID-19) (1–3). Recipients of allogeneic hematopoietic cell transplant (HCT) have about 17-fold higher risk of dying from COVID-19 than the general population (4). Post-HCT, leukocyte telomere length (TL) in recipients reflects that of their donors pre-HCT (5, 6). We examined whether the severity of COVID-19 in HCT recipients is related to leukocyte TL in the donors.

Telomeres are nucleotide-protein complexes at the ends of chromosomes. In cultured human somatic cells, telomeres shorten with each replication until they reach a critically short length, preventing further replication (7). This telomere shortening occurs in hematopoietic cells (HCs) *in vivo*, explaining age-related telomere shortening in leukocytes (8). Population-based studies are driven by the premise that a critically short TL― referred to as the ‘telomeric brink’― occurs *in vivo* in HCs (9). Thus, TL in HCs reflects their replicative history and capacity.

The relationship between severe COVID-19 and short leukocyte telomeres in the general population may be explained by a complex interplay between neutrophils (myeloid cells) and T-cells (lymphoid cells) (3). Neutrophils drive the innate immune response. They are fully differentiated myeloid cells that do not replicate once released from the bone marrow (10). T-cells, which lead the adaptive immune response, continue to replicate in extramedullary sites (11); thus leading to further telomere shortening. Therefore, T-cells have shorter telomeres than neutrophils (12).

When defending against pathogens, a T-cell response mitigates excessive tissue damage in the host by moderating the neutrophil response (13). A diminished T-cell response to SARS-CoV-2 could thus lead to an exaggerated neutrophil response, potentially resulting in pulmonary damage and severe COVID-19 (3). An unbalanced immune response to SARS-CoV-2 (14) might explain the massive neutrophil infiltrates in the lungs of patients with severe COVID-19 (15–17).

After HCT, T-cell reconstitution in recipients is critical to combatting infections (18). Many factors influence T-cell reconstitution post-HCT, including graft *versus* host disease (GVHD) and immunosuppressive therapies (18). In addition, recipients may experience a reduction in their T-cell repertoire after HCT, which might lead to impaired T-cell-mediated immune responses to specific pathogens (19–21). Thus, HCT recipients might be vulnerable to impaired T-cell responses to SARS-CoV-2 independent of T-cell TL. Accordingly, HCT provides a unique opportunity to investigate whether donor TL may contribute to an unbalanced immune response to SARS-CoV-2 in HCT recipients. This setting also excludes reverse causality, in which SARS-CoV-2 infection alters the patient’s TL.

## Methods

### Source of samples and data

Participants in this study were recipients of allogeneic HCT in remission from their original disease who were hospitalized for COVID-19 between March 2020 and January 2022. Clinical data on COVID-19 timing, treatments, illness severity, and outcomes were available from the Center for International Blood & Marrow Transplant Research (CIBMTR^®^; https://cibmtr.org/) (22), and pre-HCT blood samples for both recipients and their matched donors were available from the CIBMTR^®^ biorepository.

### COVID-19 severity and leukocyte TL measurements

We defined COVID-19 severity based on patients’ need for respiratory support: 1) mild– no respiratory distress, not requiring oxygen; 2) moderate– some respiratory distress requiring oxygen; 3) severe– respiratory distress requiring mechanical ventilation (23). We also examined the relationship between leukocyte TL parameters and HCT recipient mortality four months after COVID-19 diagnosis and hospitalization.

For this study, we included 87 allogeneic HCT recipients who had donor blood samples with high DNA integrity (5). We used Telomere Shortest Length Assay (TeSLA) to measure donors’ and recipients’ leukocyte TL in pre-HCT blood samples. TeSLA is a relatively new method of measuring TL parameters that can capture the shortest telomeres, which are usually not detected by other methods (24). TeSLA has been used recently to chart the change in leukocyte TL with age throughout the human life course (25) and in HCT recipient-donor pairs (5).

Genomic DNA was extracted from whole blood samples using the Gentra Puregene DNA Extraction Kit (Qiagen) following the manufacturer’s protocol. DNA concentration, purity, and integrity were assessed using a Nanodrop (Thermo Scientific). Extracted DNA was first ligated with single-stranded terminal adapters at telomeric overhangs, digested with four restriction enzymes (*Bfa*I, *Cvi*AII, *Ms*eI, and *Nde*I), and then ligated again to two double-stranded terminal adapters (24). The second DNA ligation improves TeSLA polymerase chain reaction’s (PCR) specificity to amplify telomeric DNA (24). Multiple TeSLA PCRs were completed, the amplified DNA was separated with 0.85% agarose gel, and then Southern Blot analysis was performed (24). Raw data from TeSLA includes band sizes for all identified leukocyte TLs (5, 24), and the output includes mean leukocyte TL and the percent of telomeres shorter than a certain threshold. The precision of TeSLA measurements, as indicated by the intraclass correlation coefficient, is 0.90 (5).We use two key TL parameters measured by TeSLA: mean TL (in kilobase [kb]) and the percentage of telomeres shorter than 3 kb.

### Statistical analysis

**Figure 1:**
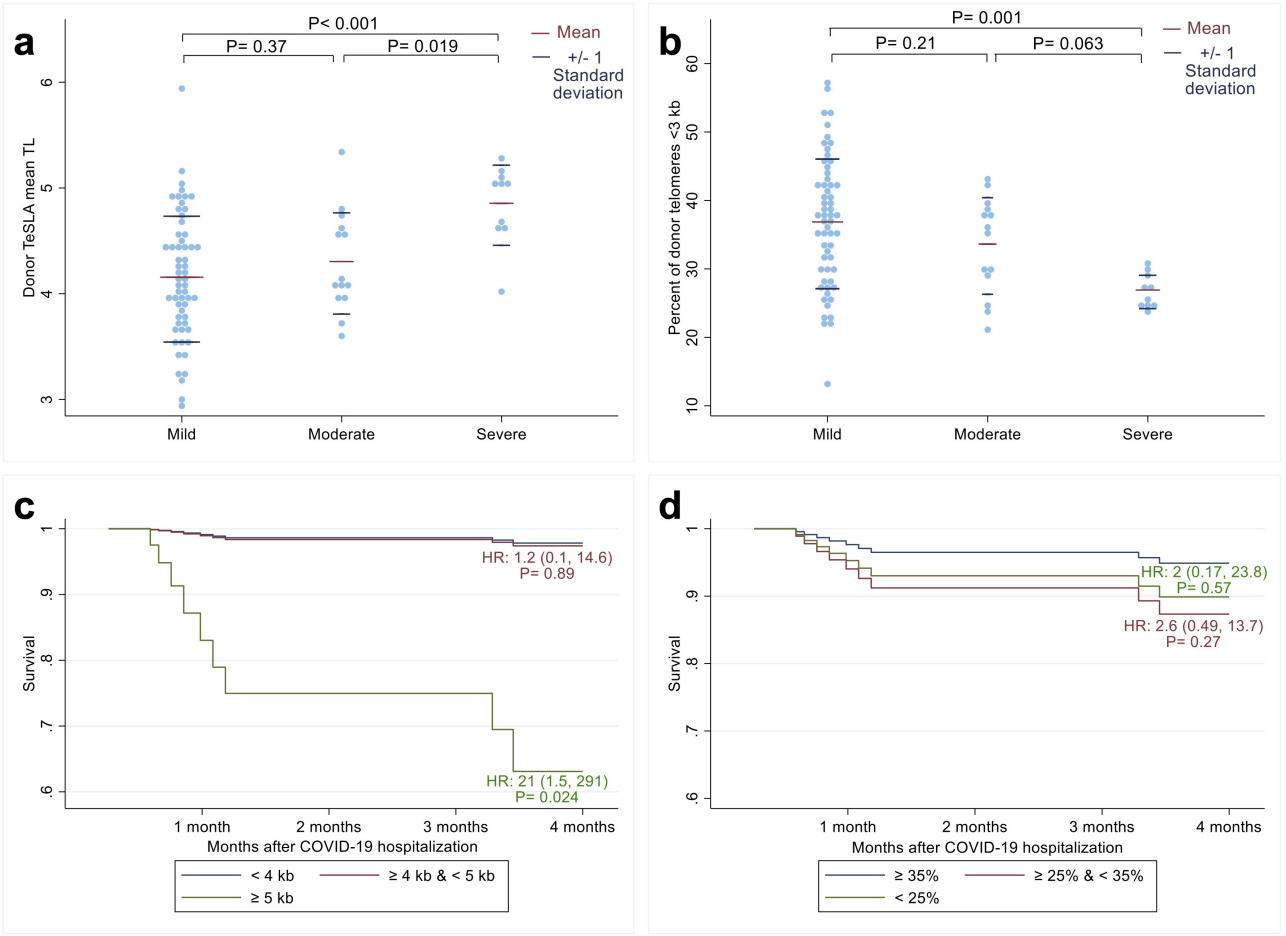
Relationship between recipients’ COVID-19 outcomes after allogeneic hematopoietic cell transplant (HCT) and donors’ leukocyte telomere length (TL) parameters before HCT. The distribution of donor mean leukocyte TL **(a)** and percent of donor telomeres shorter than 3 kilobases **(b)** among three COVID-19 disease severity groups: Mild, Moderate, and Severe (23). The p-values were calculated from t-tests. Adjusted survival probabilities after COVID-19 hospitalization among recipients of donors with mean leukocyte TL < 4 kb; ≥ 4 kb and < 5 kb; and ≥ 5kb **(c)** and among recipients of donors whose percent of telomeres shorter than 3 kb were ≥ 35%; ≥ 25% and <35%; and <25% **(d)**. HR indicates hazard ratio calculated from Cox proportional hazard model with the reference group as donor mean leukocyte TL < 4 kb (blue line) for 1c and donor percentage of short telomeres ≥ 35% (blue line) for 1d.

We compared patient characteristics and TL parameters by COVID-19 severity in three groups (mild, moderate, severe) using Fisher’s Exact test for categorical variables and the Kruskal-Wallis test for continuous variables. T-tests were used for pairwise comparisons of TL parameter differences between COVID-19 severity groups (**Figures 1a, b**).

Multinomial logistic regression models were used to test whether donors’ or recipients’ pre-alloHCT TL parameters predicted recipients’ COVID-19 severity after alloHCT. Cox regression was used to test the relationship between recipient survival and the donor or recipient leukocyte TL parameters (see **Supplementary Materials** for methods). Follow-up was available for 69 alloHCT recipients. The time scale was from COVID-19 diagnosis to follow-up. Follow-up started at the date of COVID-19 diagnosis and ended at the date of death or censoring at four months, whether participants’ COVID-19 infection status was resolved, ongoing, or improved. Two alloHCT recipients were mechanically ventilated and died at 105 days (3.4 months) and 112 days (3.7 months); we could not ascertain if their lives were artificially prolonged due to ventilation. All models were adjusted for recipient age, sex, and donor age, as these factors predict TL and (independently) predispose patients to severe COVID-19. STATA 17 software (StatCorp. TX, USA) was used for all analyses; all tests were two-sided, and p<0.05 was considered statistically significant.

## Results

HCT recipients who contracted COVID-19 were characterized as having mild (n=59), moderate (n=14), and severe (n=10) disease. On average, recipients’ age at HCT was 46 years (SD=19), and the time since HCT was 3.8 years (SD=3; **Table 1**). About 64% of HCT recipients had a history of grade III/IV acute GVHD and/or extensive chronic GVHD. Ten recipients developed severe COVID-19 at an average of 4.7 years (SD= 3.5) post-HCT. There was no difference in recipient or HCT characteristics between the three COVID-19 severity groups, except for mean recipient age at HCT (mild= 41 years, moderate= 57 years, severe= 57 years; P=0.005; **Table 1**).

**Table 1:** Characteristics of allogeneic hematopoietic cell transplant (HCT) recipients stratified by COVID-19 severity status^1^.

	Total	Mild	Moderate	Severe	
	Mean (SD) or Proportion (%)				P-value^2^
Total n	87	59	14	10	
Age (years)					
Patient (at HCT)	45.9 (19.3)	41.2 (20.2)	57.2 (11.8)	56.7 (12)	0.005
Donor (at donation)	37.1 (14)	36.5 (14.4)	43.7 (14)	30.4 (8.7)	0.096
Biological sex (Male)					
Patient	47/85 (55.3%)	33/59 (55.9%)	6/13 (46.2%)	6/9 (66.7%)	0.625
Donor	52/84 (61.9%)	38/58 (65.5%)	6/13 (46.2%)	5/9 (55.6%)	0.374
Race or ethnicity					
Patient					0.338
White	63/82 (76.8%)	42/56 (75%)	10/13 (76.9%)	9/9 (100%)	
Black or AA^3^	14/82 (17.1%)	11/56 (19.6%)	1/13 (7.7%)	0/9 (0%)	
Other	5/82 (6.1%)	3/56 (5.4%)	2/13 (15.4%)	0/9 (0%)	
Donor					0.853
White	38/64 (59.4%)	26/44 (59.1%)	4/8 (50%)	7/8 (87.5%)	
Multiple races	12/64 (18.8%)	8/44 (18.2%)	2/8 (25%)	1/8 (12.5%)	
Black or AA	7/64 (10.9%)	4/44 (9.1%)	1/8 (12.5%)	0/8 (0%)	
Other	7/64 (10.9%)	6/44 (13.6%)	1/8 (12.5%)	0/8 (0%)	
Disease indication for HCT^4^					0.154
AML	22/83 (26.5%)	16/56 (28.6%)	3/14 (21.4%)	2/9 (22.2%)	
MDS	18/83 (21.7%)	8/56 (14.3%)	6/14 (42.9%)	3/9 (33.3%)	
ALL	13/83 (15.7%)	10/56 (17.9%)	0/14 (0%)	3/9 (33.3%)	
NHL	7/83 (8.4%)	5/56 (8.9%)	1/14 (7.1%)	0/9 (0%)	
MPN	6/83 (7.2%)	3/56 (5.4%)	1/14 (7.1%)	1/9 (11.1%)	
Other^5^	17/83 (20.5%)	14/56 (25%)	3/14 (21.4%)	0/9 (0%)	
Years since HCT	3.8 (3)	3.8 (3.2)	3.1 (2.5)	4.7 (3.5)	0.48
Acute graft *versus* host disease (GVHD)					0.918
None	33/87 (37.9%)	24/59 (40.7%)	4/14 (28.6%)	4/10 (40%)	
Grade 1	15/87 (17.2%)	9/59 (15.2%)	4/14 (28.6%)	2/10 (20%)	
Grade 2	27/87 (31%)	18/59 (30.5%)	5/14 (35.7%)	3/10 (30%)	
Grade 3	12/87 (13.8%)	8/59 (13.6%)	1/14 (7.1%)	1/10 (10%)	
Chronic GVHD					0.128
None/Limited	39/87 (44.8%)	30/59 (50.8%)	3/14 (21.4%)	4/10 (40%)	
Extensive	48/87 (55.2%)	29/59 (49.2%)	11/14 (78.6%)	6/10 (60%)	
COVID-19 Treatment					
Corticosteroids	23/82 (28.1%)	6/58 (10.3%)	7/14 (50%)	10/10 (100%)	<0.001
Antivirals	24/33 (72.7%)	7/13 (53.9%)	8/10 (80%)	9/10 (90%)	0.18
Initial lab values					
WBCs (109/L; n=56)	7.25 (5.27)	6.11 (3.1)	10.8 (8.5)	7.7 (4.9)	0.039
Lymphocytes (%, n=53)	21.4 (15)	23.9 (13.9)	17.4 (19.7)	16.4 (11)	0.071
Neutrophils (%, n=53)	65.5 (19.8)	61.5 (19.9)	74 (18.9)	70.6 (18.2)	0.079
Pre-HCT TeSLA mean LTL^6^ (kb)					
Patient	3.7 (0.82)	3.8 (0.87)	3.6 (0.75)	3.7 (0.48)	0.78
Donor	4.3 (0.59)	4.2 (0.59)	4.3 (0.48)	4.9 (0.38)	0.001
Pre-HCT TeSLA percent of LTL <3kb (%)					
Patient	44.7 (14.7)	43.9 (15.5)	45 (14)	44.5 (9.5)	0.96
Donor	35.2 (9)	36.9 (9.5)	33.6 (7.1)	26.9 (2.4)	0.002

^1^Mild, no oxygenation needed; Moderate, oxygen needed, but not mechanical ventilation; Severe, mechanical ventilation needed.

^2^Kruskal-Wallis and Fisher’s Exact test statistics were conducted.

^3^AA, African American.

^4^AML, Acute myeloid leukemia; MDS, Myelodysplastic diseases; ALL, Acute lymphoblastic leukemia; NHL, Non-Hodgkin lymphoma; MPN, Myeloproliferative diseases.

^5^Other includes Hodgkin lymphoma; severe aplastic anemia; disorders of the immune system; plasma cell disease; inherited disorders of metabolism; inherited abnormalities of erythrocyte differentiation or function; inherited bone marrow failure syndromes; histiocytic disease; hemoglobinopathies; chronic myeloid leukemia.

^6^LTL, Leukocyte telomere length.

In unadjusted models, long donor leukocyte TL was associated with more severe disease, with a mean TL difference of 551 base pairs (bp) between patients with severe compared to moderate disease (P=0.019) and 699 bp between patients with severe compared to mild disease (P<0.001; **Figure 1a**). In line with these findings, patients with severe COVID-19 had donors with a lower percentage of telomeres shorter than 3 kb than patients with moderate COVID-19 (mean difference=-6.7%; 95% CI: -13.8%, 0.37%; P=0.063) and mild COVID-19 (mean difference= -9.9%; 95% CI: -15.8%, -4.1%; P=0.001; **Figure 1b**).

Multivariable analyses confirmed these findings (**Supplementary Table S1**), showing that the relative risk of patients having severe compared to mild COVID-19 was 148 times higher for each kb increase in donor mean TL (95% CI: 4.5-4867, P=0.005) and 0.8 times lower for each percent increase in donor TL shorter than 3 kb (95% CI: 0.68-0.95; P=0.01). The relative risk of patients having moderate COVID-19 compared to mild COVID-19 was 3.9 times higher for each kb increase in donors’ mean TL (95% CI: 0.8, 18.8; P=0.09) and 0.89 times higher for each percent decrease in donors’ TL shorter than 3 kb (95% CI: 0.8, 0.997; P=0.04).

In survival analysis (**Supplementary Table S2**), each kb increase in pre-HCT donors’ mean leukocyte TL was associated with a 9.7 times mortality hazard among HCT recipients (95% CI: 1.2, 77.2; P=0.03). HCT recipients who had donors with mean TeSLA TL ≥ 5 kb had a 21 times higher risk of mortality (95% CI= 1.5, 291; P=0.024) compared to recipients who had HCT donors with mean TL < 4 kb (**Figure 1c**). However, we found no statistically significant associations between patient survival and the percentage of donors’ telomeres shorter than 3 kb (**Figure 1d**).

We found no relationship between recipients’ pre-HCT TL parameters and COVID severity or 4-month survival (**Supplementary Tables S3**, **S4**).

## Discussion

Our study shows that recipients of HCT from donors with long leukocyte telomeres are at a higher risk of respiratory distress and mortality upon SARS-CoV-2 infection. These results contrast with findings from the general population, showing that adults with long leukocyte telomeres are less likely to have severe COVID-19 (1–3). We propose that severe COVID-19 in both populations might be due, in part, to an unbalanced neutrophil and T-cell response but with different TL-related mechanisms.

**Figure 2:**
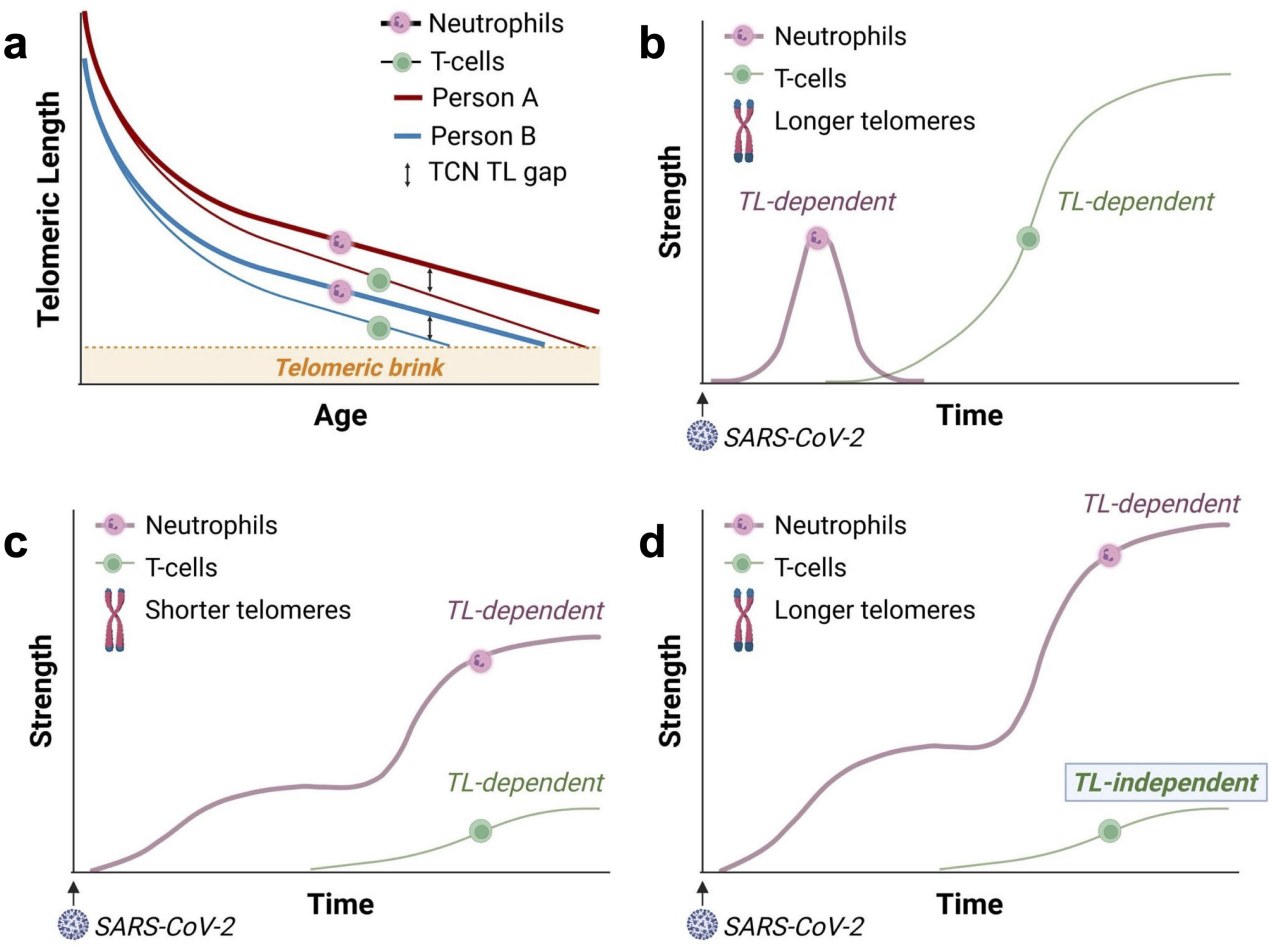
The growing T-cell-neutrophil (TCN) telomere length (TL) gap with age and the responses of T-cells and neutrophils to SARS-CoV-2. “Strength” on the y-axis refers to the strength of the leukocyte response. **(a)** Telomeres shorten faster in T-cells than neutrophils, leading to a widening TCN TL gap with age (12). Consequently, in adults, particularly older adults or adults with short telomeres, the TL-dependent replicative capacity of T-cells is significantly reduced compared to that of myelocytes— the precursors of neutrophils. Due to inter-individual heterogeneity, person A is born with longer telomeres than person B and, thus, reaches the telomeric brink later in life. The “telomeric brink” denotes the zone of critically short telomeres halting further replication. **(b)** In young adults with longer leukocyte TL, the initial neutrophil response (reflecting innate immunity) is moderated by the build-up of the adaptive T-cell response. This results in a coordinated (balanced), TL-dependent immune response involving neutrophils and T-cells. The delayed T-cell response reflects the time required to generate SARS-CoV-2-specific effector T-cells through clonal expansion. Panels c – d display schematic representations of the responses of neutrophils and SARS-CoV-2-specific T-cells in COVID-19, based on Sette & Crotty’s framework (14). **(c)** In older adults and individuals who inherit short telomeres, a weak, TL-dependent T-cell response fails to adequately moderate the neutrophil response, leading to an intense (unbalanced) neutrophil response. **(d)** For recipients of allogeneic HCT, a weak T-cell response is likely due to diminished diversity in the T-cell repertoire, which is independent of TL. In HCT recipients from donors with long telomeres, the weak T-cell response unleashes a massive TL-dependent neutrophil response, surpassing that observed in individuals from the general population **(c)**.

While TL is highly variable across individuals (9, 12, 25), TL is strongly correlated among different types of leukocytes, including myeloid and lymphoid cells, within an individual (26). However, as leukocyte telomeres shorten with age, the TL difference between T-cells and neutrophils within an individual increases. Unlike neutrophils, T-cells continue replicating after their release from the bone marrow. This results in a widening gap between the length of telomeres in T-cells and neutrophils as people age (12). We refer to it as the “T cell-neutrophil” (TCN) gap (**Figure 2a**). The TCN TL gap indicates that myelocytes, the bone marrow precursors of neutrophils, have more TL-dependent replicative capacity than T-cells, and this difference is larger in older people who generally have shorter telomeres than in younger people.

As the T-cell response mitigates the neutrophil response (13), a weak TL-dependent T-cell response to SARS-CoV-2 may unleash an excessive neutrophil response, causing pulmonary damage, respiratory distress, and severe COVID-19. This unbalanced, TL-dependent immune response (**Figures 2b, c**), which is based on the framework outlined by Sette and Crotty (14), is supported by a recent study linking T-cell lymphopenia (low lymphocyte count) with short T-cell telomeres in patients with COVID-19 (27). Thus, adults with short leukocyte telomeres due to age or genetic factors might be at a higher risk for severe COVID-19. Findings from the UK Biobank COVID-19 study support this explanation (1). In the UK Biobank study, leukocyte TL was measured years before the pandemic, thus excluding the possibility of reverse causation, where short leukocyte telomeres are a consequence of COVID-19.

In the context of allogeneic HCT, recipients’ leukocyte TL reflects that of their donors, regardless of recipient age (5, 6). While long telomeres post-HCT could theoretically enhance immune responses to SARS-CoV-2, the reduction in T-cell repertoire following HCT (19–21) might weaken the T-cell response, independent of TL (28). Our model suggests that recipients of HCT from donors with longer leukocyte TL experience a weak T-cell response to SARS-CoV-2 independent of TL. This inadequate response unleashes a massive TL-dependent neutrophil response (**Figure 2d**).

Thus, while the weak T-cell response in older individuals in the general population may reflect their short telomeres (**Figure 2c**), in HCT recipients, a weak T-cell response is likely TL-independent due to HCT-related impaired adaptive immunity (**Figure 2d**). However, the myelocyte response (and thus the neutrophil response) in HCT recipients remains TL-dependent. By this logic, when contracting COVID-19, a weak, TL-independent T-cell response would poorly mitigate a massive TL-dependent neutrophil response in recipients of HCT from donors with long leukocyte telomeres (**Figure 2d**).

We also note that immunosuppressive therapy for GVHD (29) might weaken the T-cell response (18), independent of TL. We cannot exclude this possibility, although we found no significant association between GVHD and COVID-19 severity (**Table 1**). Nevertheless, the conclusion that an unbalanced immune response in HCT recipients from donors with long telomeres holds regardless of whether the cause is diminished T-cell repertoire, immunosuppressive therapy, or both.

Finally, we acknowledge that our study is small and cannot prove causality. Larger studies and functional evaluation studies are warranted to test our proposed model and recommend implications to the field of HCT. Research is also needed to understand further the relationship between diminished T-cell repertoire and TL-dependent T-cell replicative capacity post-HCT.

In conclusion, we propose a model where allogeneic HCT recipients experience a TL-independent, weak T-cell response that results in an excessive TL-dependent neutrophil response to SARS-CoV-2. The outcome is severe lung damage and respiratory distress, particularly in recipients of HCT from donors with long leukocyte telomeres. If our findings are confirmed, allogeneic HCT could serve as a human experimental model, providing valuable insights into the role of telomeres in the balance between the innate and adaptive immune responses against pathogens.

## Data Availability

The datasets presented in this article are not readily available because deidentified data from this study are available upon request. Data access permission will require a material transfer agreement. Requests to access the datasets should be directed to Shahinaz Gadalla, shahinaz.gadalla@nih.gov.
