# Supplementary material for: Long donor leukocyte telomeres raise risk of severe COVID-19 in recipients of allogeneic hematopoietic cell transplant
Source: Front Immunol. 2025 Apr 29;16:1524608. doi: 10.3389/fimmu.2025.1524608 (PMC12069395; doi:10.3389/fimmu.2025.1524608)
Supplement: Supplementary file 1 [file Table1.docx]

Supplemental Materials

**Supplemental Table S1.** Multinomial logistic regression model (n=80*) examining adjusted relationship between recipients’ COVID-19 severity after allogeneic hematopoietic cell transplant (HCT; dependent variable) and donors’ leukocyte telomere length (LTL) parameters before HCT (independent variable).

|  | Relative Risk Ratio (95% CI) | P-value |
| --- | --- | --- |
| **Donor mean LTL** | | |
| **Mild COVID-19** | REF | |
| **Moderate COVID-19** |  | |
| Donor mean LTL | 3.88 (0.8, 18.8) | 0.093 |
| Patient age | 1.06 (1.01, 1.11) | 0.026 |
| Donor age | 1.05 (0.99, 1.11) | 0.11 |
| Patient sex | 0.77 (0.2, 2.9) | 0.7 |
| **Severe COVID-19** |  | |
| Donor mean LTL | 148.5 (4.53, 4867) | 0.005 |
| Patient age | 1.11 (1.02, 1.2) | 0.012 |
| Donor age | 1.02 (0.94, 1.11) | 0.67 |
| Patient sex | 1.31 (0.2, 8.5) | 0.78 |
| **Percentage (%) of donor telomeres < 3 kb** | | |
| **Mild COVID-19** | REF | |
| **Moderate COVID-19** |  | |
| Percentage of donor telomeres <3 kb | 0.89 (0.8, 0.997) | 0.044 |
| Patient age | 1.06 (1.01, 1.11) | 0.029 |
| Donor age | 1.06 (0.996, 1.13) | 0.069 |
| Patient sex | 0.87 (0.23, 3.4) | 0.85 |
| **Severe COVID-19** |  | |
| Percentage of donor telomeres <3 kb | 0.8 (0.68, 0.95) | 0.01 |
| Patient age | 1.07 (1.01, 1.14) | 0.017 |
| Donor age | 1.01 (0.93, 1.09) | 0.88 |
| Patient sex | 1.8 (0.32, 9.9) | 0.5 |

*n=2 were missing data on patient age, donor age, and patient sex; n=1 was missing data on donor age; and n=4 were missing data on oxygenation status.

**Supplemental Table S2.** Cox proportional hazards regression models (n=67*) examining adjusted relationship between HCT recipients’ 4-month survival after hospitalization for COVID-19 and donors’ leukocyte telomere length (LTL) parameters before HCT (independent variable).

|  | Hazard ratio (95% CI) | P-value |
| --- | --- | --- |
| **Donor mean LTL** | | |
| **Mean LTL (continuous)** | 9.7 (1.2, 77.2) | 0.032 |
| Patient age | 1.08 (1.02, 1.14) | 0.011 |
| Donor age | 0.98 (0.91, 1.05) | 0.56 |
| Patient sex | 1.06 (0.25, 4.5) | 0.94 |
| **Mean LTL (categories)** |  |  |
| < 4 kb | REF |  |
| ≥ 4 kb & < 5 kb | 1.2 (0.1, 14.6) | 0.89 |
| ≥ 5 kb | 20.9 (1.5, 291) | 0.024 |
| Patient age | 1.11 (1.02, 1.2) | 0.016 |
| Donor age | 0.98 (0.9, 1.07) | 0.64 |
| Patient sex | 1.01 (0.23, 4.42) | 0.99 |
| **Percentage (%) of donor telomeres < 3 kb** | | |
| **Percentage of telomeres < 3kb (continuous)** | 0.92 (0.83, 1.03) | 0.14 |
| Patient age | 1.06 (1.01, 1.12) | 0.019 |
| Donor age | 0.96 (0.9, 1.04) | 0.32 |
| Patient sex | 1.43 (0.35, 5.8) | 0.62 |
| **Percentage of telomeres < 3kb (categories)** |  |  |
| ≥ 35% | REF |  |
| ≥ 25% & <35% | 2.58 (0.49, 13.7) | 0.27 |
| < 25% | 2.03 (0.17, 23.8) | 0.57 |
| Patient age | 1.06 (1.003, 1.11) | 0.039 |
| Donor age | 0.96 (0.89, 1.03) | 0.26 |
| Patient sex | 1.44 (0.35, 5.9) | 0.61 |

*n=1 was missing data on patient age, donor age, and patient sex; n=1 was missing data on donor age.

**Supplemental Table S3.** Multinomial logistic regression models (n=77* examining unadjusted relationship between HCT recipients’ COVID-19 severity (dependent variable) and recipients’ leukocyte telomere length (LTL) parameters before HCT (independent variable).

|  | Relative Risk Ratio (95% CI) | P-value |
| --- | --- | --- |
| **Recipient mean LTL** | | |
| Mild COVID-19 | REF | |
| Moderate COVID-19 | 0.75 (0.35, 1.6) | 0.45 |
| Severe COVID-19 | 0.84 (0.34, 2.05) | 0.7 |
| **Percentage (%) of recipient telomeres < 3 kb** | | |
| Mild COVID-19 | REF | |
| Moderate COVID-19 | 1.01 (0.97, 1.05) | 0.8 |
| Severe COVID-19 | 1 (0.96, 1.05) | 0.91 |

*n=4 were missing data on oxygenation status.

**Supplemental Table S4.** Cox proportional hazards regression models (n=64*) examining unadjusted relationship between HCT recipients’ 4-month survival after hospitalization for COVID-19 and recipients’ leukocyte telomere length (LTL) parameters before HCT (independent variable).

|  | Hazard ratio (95% CI) | P-value |
| --- | --- | --- |
| **Recipient mean LTL** | | |
| Mean LTL (continuous) | 0.74 (0.34, 1.62) | 0.46 |
| **Percentage (%) of recipient telomeres < 3 kb** | | |
| Percentage of telomeres < 3kb (continuous) | 1.01 (0.97, 1.06) | 0.51 |

*n=17 were missing data on follow-up time.
